# Autophagy and Cellular Senescence in Alzheimer's Disease: Key Drivers of Neurodegeneration

**DOI:** 10.1111/cns.70503

**Published:** 2025-07-23

**Authors:** Md Sadique Hussain, Neetu Agrawal, Baby Ilma, Rekha M M, Priya Priyadarshini Nayak, Mandeep Kaur, Anil Khachi, Kavita Goyal, Arcot Rekha, Saurabh Gupta, Gaurav Gupta, Kamal Dua

**Affiliations:** ^1^ Uttaranchal Institute of Pharmaceutical Sciences Uttaranchal University Dehradun India; ^2^ School of Pharmaceutical Sciences Lovely Professional University Phagwara Punjab India; ^3^ Institute of Pharmaceutical Research GLA University Mathura Uttar Pradesh India; ^4^ Sharda School of Pharmacy Sharda University Greater Noida Uttar Pradesh India; ^5^ Department of Chemistry and Biochemistry, School of Sciences JAIN (Deemed to Be University) Bangalore Karnataka India; ^6^ Department of Medical Oncology, IMS and SUM Hospital Siksha ‘O’ Anusandhan (Deemed to Be University) Bhubaneswar Odisha India; ^7^ Department of Allied Healthcare and Sciences Vivekananda Global University Jaipur Rajasthan India; ^8^ Department of Applied Sciences, Chandigarh Engineering College Chandigarh Group of Colleges‐Jhanjeri Mohali Punjab India; ^9^ Department of Biotechnology Graphic Era (Deemed to Be University) Dehradun India; ^10^ Dr.D.Y.Patil Medical College, Hospital and Research Centre Pimpri, Pune India; ^11^ Department of Pharmacology Chameli Devi Institute of Pharmacy Indore Madhya Pradesh India; ^12^ Centre for Research Impact & Outcome, Chitkara College of Pharmacy Chitkara University Rajpura Punjab India; ^13^ Centre of Medical and Bio‐Allied Health Sciences Research Ajman University Ajman UAE; ^14^ Discipline of Pharmacy, Graduate School of Health University of Technology Sydney Sydney New South Wales Australia; ^15^ Woolcock Institute of Medical Research Macquarie University Sydney New South Wales Australia

**Keywords:** aging brain, neurodegeneration, neuroinflammation, senolytics

## Abstract

**Background:**

Alzheimer's disease (AD) is a progressive neurodegenerative disorder in the elderly, characterized by extracellular amyloid β‑ (Aβ) plaque deposition and intracellular neurofibrillary tangles (NFTs). Impaired autophagy, the cellular pathway for degrading damaged organelles and misfolded proteins, and cellular senescence, permanent cell cycle arrest with proinflammatory secretions, have emerged as key contributors to AD pathogenesis.

**Methods:**

We performed a narrative review of recent mechanistic and preclinical studies investigating (1) autophagic flux and its role in Aβ and tau clearance; (2) the accumulation and secretory phenotype of senescent cells in the aging brain; (3) interactions between autophagy impairment and senescence; and (4) the efficacy of autophagy enhancers (e.g., rapamycin and metformin) and senolytic agents in rodent models of AD.

**Results:**

Defective autophagosome–lysosome fusion in AD causes autophagic vacuole buildup with amyloid precursor protein and β‑secretase, boosting Aβ generation and hindering tau clearance, promoting neurofibrillary tangles. In AD models, senescent neurons and microglia release pro‑inflammatory cytokines (SASP), fueling neuroinflammation and synaptic dysfunction. Decline in autophagy induces senescence and blocks clearance in a vicious cycle. Rapamycin and metformin restore autophagic flux, reduce Aβ and tau pathologies, and improve memory. Senolytics clear senescent cells, reduce inflammation, and rescue cognition.

**Conclusion:**

Dysregulated autophagy and cellular senescence interact to drive the progression of AD. Targeting these pathways with autophagy‐boosting drugs and senolytic agents holds promise for disease‐modifying therapies aimed at halting or reversing neurodegeneration in Alzheimer's disease.

## Graphical abstract

Impaired autophagy in Alzheimer's disease leads to accumulation of amyloid‐β and tau aggregates, while senescent brain cells secrete pro‐inflammatory SASP factors. The interplay between defective clearance and chronic inflammation accelerates neuronal dysfunction and highlights autophagy enhancers and senolytics as promising therapeutic strategies.

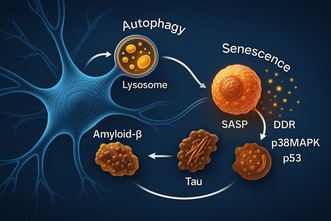

## 1 Introduction

Alzheimer's disease (AD) is the predominant type of dementia. A decline in cognitive abilities, functioning, and behavior characterizes it. The first symptom is usually a decline in memory related to current events [1]. The defining clinical characteristics seen in the brain cells of individuals with AD are elevated concentrations of both amyloid‐β (Aβ), which forms extracellular senile plaques, and hyperphosphorylated tau (hTau), which aggregates intracellularly as neurofibrillary tangles (NFTs) [2, 3]. Approximately 50 million individuals worldwide are already affected by dementia. This data is expected to triple by 2050 owing to the elderly population, leading to a higher likelihood of impairment, increased burden of sickness, and rising healthcare expenses [4, 5]. Furthermore, existing therapeutic approaches alleviate signs, and there is no viable treatment for AD.

Nevertheless, AD has a lengthy prodromal phase, throughout which early intervention seems to be especially crucial to decelerate the advancement of AD. Consequently, epidemiological research is necessary to discover the risk and safeguarding factors that significantly impact cognitive state. Approximately 33% of AD instances globally may be ascribed to identifiable variables influencing an individual's likelihood of getting AD [6]. Aging is an unavoidable occurrence in the lifespan of a living being, characterized by physical decline and heightened susceptibility to illness and mortality [7]. The pace of degradation in the process of aging varies across different animals, people, and cells [8]. With the rise in the number of elderly individuals, there is a corresponding rise in the economic strain caused by age‐related diseases (ARDs). Consequently, it is imperative to prioritize efforts to postpone or avoid the onset of ARDs. Neurodegenerative disorders (NDs) and related cognitive impairments are just two of the many illnesses that older people face; these conditions have an impact on their living standards and healthy lives. Neurodegeneration is a complex cerebral condition that remains incompletely understood. Aging indicators are the most significant risk factors related to neurodegeneration [9, 10].

**FIGURE 1:**
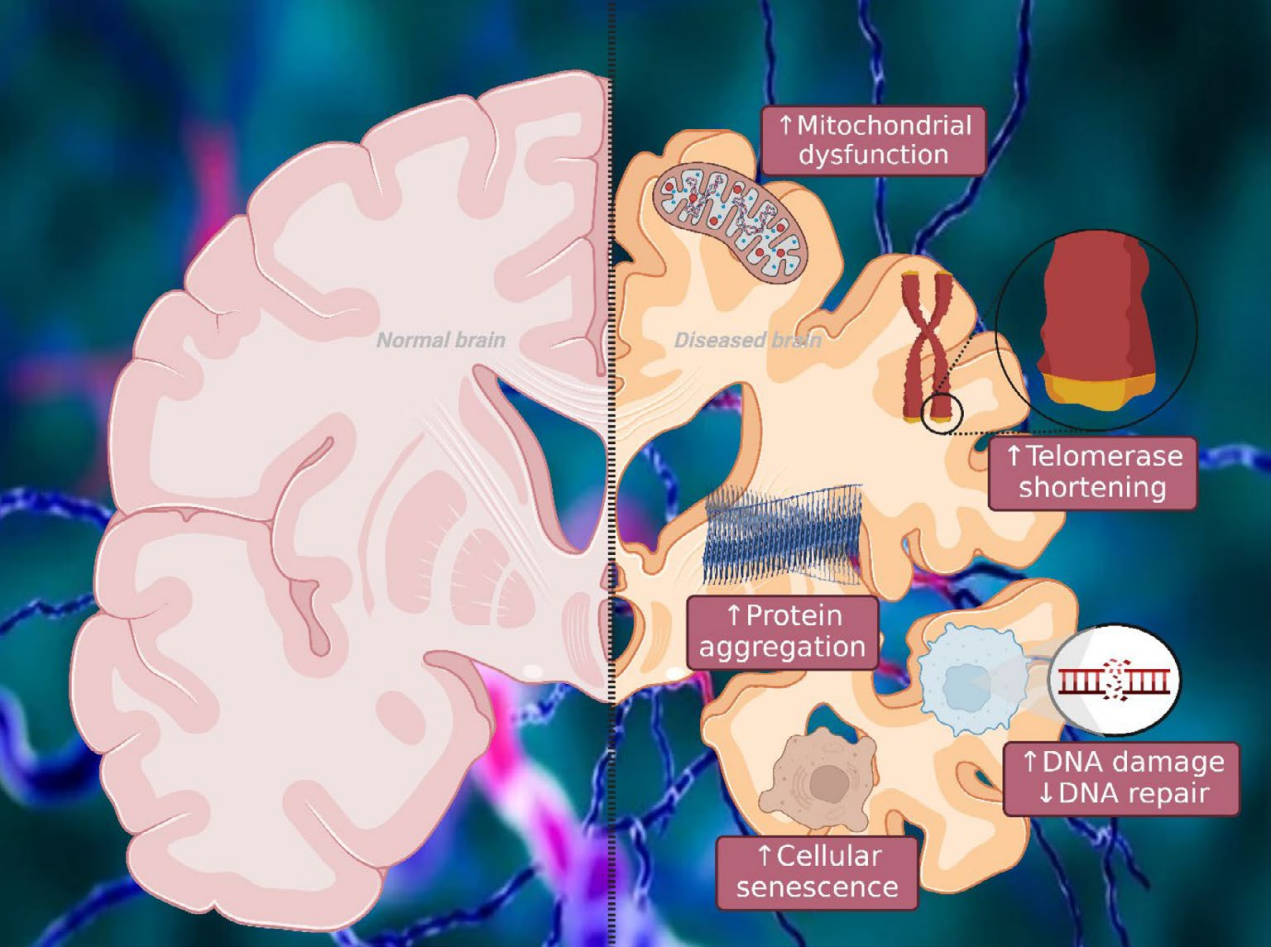
Characteristics of brain aging and its association with Alzheimer's disease. Aging results from numerous disturbances in the body's balance, such as the accumulation of proteins, DNA damage, malfunctioning of mitochondria, dysfunction of lysosomes, and alterations in epigenetic control. The occurrence of these modifications is contingent upon the specific cell types involved, leading to the emergence of diverse illnesses that vary based on their beginnings within distinct brain regions and how they spread.

The characteristic features of aging, such as the elimination of impaired mitochondria, the loss of cell function, the deterioration of genetic material, and the presence of misfolded proteins, have been associated with many NDs, including AD (Figure 1) [7]. The relationship between ARDs in the brain and the onset of neurodegeneration is uncertain. Furthermore, the two most widespread NDs, AD and Parkinson's disease (PD), have the shared characteristic of protein accumulation, albeit with their distinct clinical signs. The buildup of senile plaques, which comprise extracellular Aβ, and the development of intraneuronal tau‐containing NFTs are significant pathological characteristics of AD. Similarly, misfolded α‐synuclein (α‐syn) buildup is an essential pathogenic sign of PD [11]. Amyotrophic lateral sclerosis (ALS) and frontotemporal lobar dementia (FTD) both exhibit an accumulation of proteins. In ALS, the 43‐kDa TAR DNA‐binding protein (TDP‐43) clumps, whereas in FTD and AD, the microtubule‐associated protein tau (MAPT) accumulates [12, 13, 14].

Autophagy and cellular senescence are vital cellular processes that significantly influence neurodegeneration, a complex disorder marked by the progressive loss of neurons and their function. Autophagy is an innate cellular process that involves the recycling and eliminating of impaired or malfunctioning cellular parts, such as proteins and organelles. Its purpose is to ensure cellular balance and avoid the buildup of harmful substances [15, 16]. Autophagy has been linked to removing protein clumps and other toxic substances that have a role in NDs within neurodegeneration. Autophagy has been demonstrated to have a preventive function in removing α‐syn and Aβ [17, 18, 19]. Autophagy also serves to preserve the integrity and functionality of mitochondria, which is essential for the ongoing existence of neurons. Many NDs are characterized by mitochondrial dysfunction. Autophagy aids in eliminating damaged mitochondria and preserving mitochondrial balance, hence mitigating the risk of neurodegeneration [20].

**FIGURE 2:**
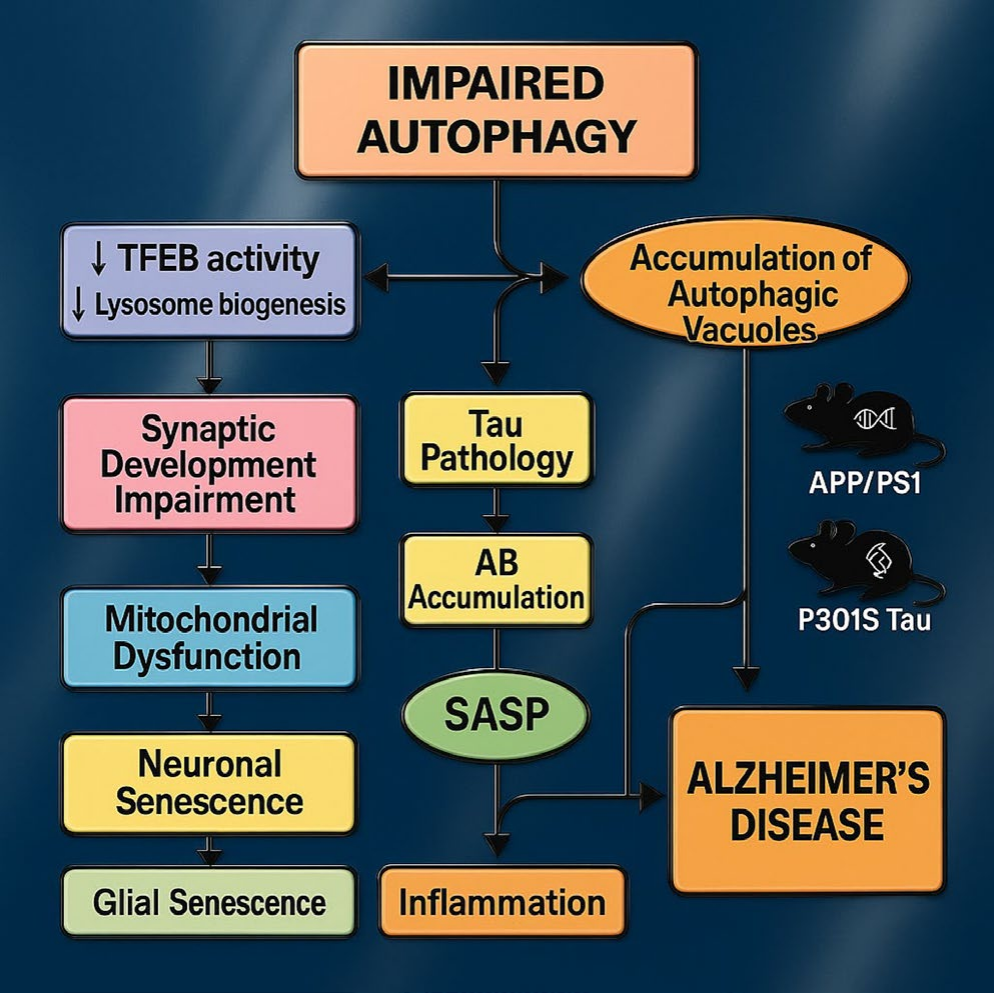
Impaired autophagy–senescence axis in Alzheimer's disease. This schematic illustrates how defective autophagy driven by reduced TFEB activity and lysosome biogenesis leads to accumulation of autophagic vacuoles and initiates tau pathology alongside Aβ buildup. The resulting SASP promotes neuronal and glial senescence, mitochondrial dysfunction, synaptic impairment, and chronic inflammation, collectively fueling Alzheimer's pathology. APP/PS1 and P301S Tau mouse icons denote the key preclinical models used to study these interconnected mechanisms.

Cellular senescence is when cells permanently stop dividing due to many types of cellular stressors, such as damage to DNA, oxidative stress (OS), and telomere reduction. Senescent cells (SCs) may progressively amass as an individual ages and have a role in the progression of ARDs. Within neurodegeneration, SCs can potentially exacerbate disease advancement by releasing pro‐inflammatory substances, such as the senescence‐associated secretory phenotype (SASP), which may incite inflammation and harm tissues [21]. SCs may also hinder the growth and specialization of stem cells, leading to a decline in the functionality of neural stem cells (NSCs) and a deterioration in the preservation of brain tissue [22, 23]. Autophagy and cellular senescence are interrelated phenomena that may mutually impact one another's activities. Autophagy, for instance, aids in the elimination of impaired organelles and proteins that could trigger cellular senescence (Figure 2).

In contrast, SCs can hinder autophagy, resulting in the buildup of impaired cellular elements and worsening the neurodegeneration process [17, 20]. The recent literature emphasizes the relevance of faltered autophagy and cellular senescence in AD progression. This combination of impaired autophagic activity with an exacerbated state of senescence speeds up the neurodegenerative process and cognitive deterioration more drastically. Studying these mechanisms brings a deep understanding of the molecular basis of AD and potential therapeutic targets. This study provides a further understanding of the complex relationship between autophagy dysfunction, SCs, and AD with the ultimate goal of improving therapeutic strategies that can reduce the disease load posed by AD upon an aging global population.

## 2 Autophagy in the Brain

Autophagy is essential to sustaining equilibrium in cells and controlling the integrity of proteins by effectively breaking down and recycling defective or broken cellular components. The procedure entails the creation of autophagosomes, which capture and convey defective organelles and proteins to lysosomes for breakdown and recycling [24, 25, 26]. Numerous processes govern autophagy in NDs, including chaperone‐mediated autophagy (CMA). CMA specifically directs substrates containing the KFERQ motif toward destruction by lysosomes. The cognitive mental ability is compromised in NDs, resulting in the buildup of disease‐causing proteins. Studies suggest that improving CMA by genetic and pharmacological methods may enhance the breakdown of proteins linked with NDs and reduce the symptoms of these diseases [27, 28]. Aside from CMA, lysosomal pathways such as Transient Receptor Potential Channel Mucolipin 1 (TRPML1) and Two‐Pore Channel isoform 2 (TPC2) notably impact lysosomal balance and autophagy. Malfunction of these routes involves abnormal autophagy and the gradual death of nerve cells, and it has been suggested that manipulating TRPML1 and TPC2 with drugs might be a potential treatment approach to restore proper autophagy processes in NDs [29, 30].

Hypoxia, a frequent characteristic of NDs, is crucial in modulating autophagy via many signaling networks. Hypoxia may have contrasting effects on autophagy, either promoting or inhibiting it, and its abnormal modulation might trigger NDs [31]. Additionally, microglia also have a vital function in controlling autophagy and neuroinflammation. Microglial autophagy helps break down damaged proteins and other toxic substances neurons create. When this process is not regulated correctly, it is associated with NDs [32, 33]. Moreover, scientific studies have demonstrated that ketone bodies and the ketogenic diet effectively control autophagy by modulating many cellular mechanisms. These actions include improving the functioning of mitochondria, decreasing OS, and regulating changes in the structure and functioning of histones and non‐histone proteins via epigenetic and post‐translational modifications [34].

Echinacoside, a naturally occurring phenylethanoid glycoside, has been shown to have neuroprotective properties via the regulation of autophagy, preservation of mitochondrial activity, and reduction of OS [35]. Furthermore, long non‐coding RNAs (lncRNAs) have been linked to the control of autophagy in NDs. Dysregulated control of lncRNAs is a contributing factor to neurological illnesses. Investigating these lncRNAs may provide valuable knowledge on developing and identifying NDs [36, 37]. Gaining an in‐depth comprehension of these processes and systems is of utmost importance in creating treatment options that effectively control autophagy and address NDs.

### 2.1 Autophagy in Synapse Development

Neuronal autophagy (NAg) is crucial for the development of synapses. Research has shown that manipulating autophagy levels by lowering or elevating them leads to proportional changes in synapse development [38]. The researchers discovered that NAg had a beneficial effect on synaptic formation in the Drosophila neuromuscular intersection. Inducing elevated amounts of autophagy and enhancing synaptic development may be achieved by overexpressing Atg1, a serine/threonine kinase that is a crucial autophagy modulator. Conversely, when autophagy function is decreased due to alterations in autophagy genes, the dimension of synapses is reduced. The ubiquitin‐proteasome system (UPS) is recognized as a significant inhibitory controller of synaptic growth at the neuromuscular intersection [39]. NAg and the UPS work together to control the formation of synapses by controlling Hiw, an E3 ubiquitin (Ub) ligase. It has been reported that Atg1 regulates synaptic formation by reducing the expression of the MAP kinase ERK [40]. Mice with an Atg1 mutant saw reduced synaptic density due to heightened ERK action, indicating that active ERK inhibits synapse development [41, 42]. Given that autophagy is triggered via numerous evolutionary and environmental signals, it is plausible that NAg is crucial for promoting the growth and adaptability of synapses, which are crucial for memory and cognition processes.

### 2.2 Autophagy in Injured Neurons

The axon is a unique and efficient component designed explicitly for transmitting signals across long distances inside the neurological system. Prior research has shown that autophagy may be linked to axonal degeneration after axotomy or excitotoxic stimulation. Following axotomy, there is a buildup of autophagosome‐like vesicles (ALVs) at the axon terminals [43, 44]. The augmented autophagy is followed by chromatolysis, a cytoplasmic region lacking organelles and containing several vesicles, notably autophagosomes [43]. In the Purkinje cells of Lurcher mice, which are used as a model for excitotoxic neurodegeneration, the buildup of autophagosomes tagged with GFP‐LC3 in dystrophic axonal swellings is triggered by excitotoxic shocks. This buildup is a characteristic feature of CNS axonopathy. ALVs have been seen in impaired axons in AD, PD, and Huntington's disease (HD) [45, 46, 47, 48, 49, 50, 51].

## 3 Autophagy in AD

**FIGURE 3:**
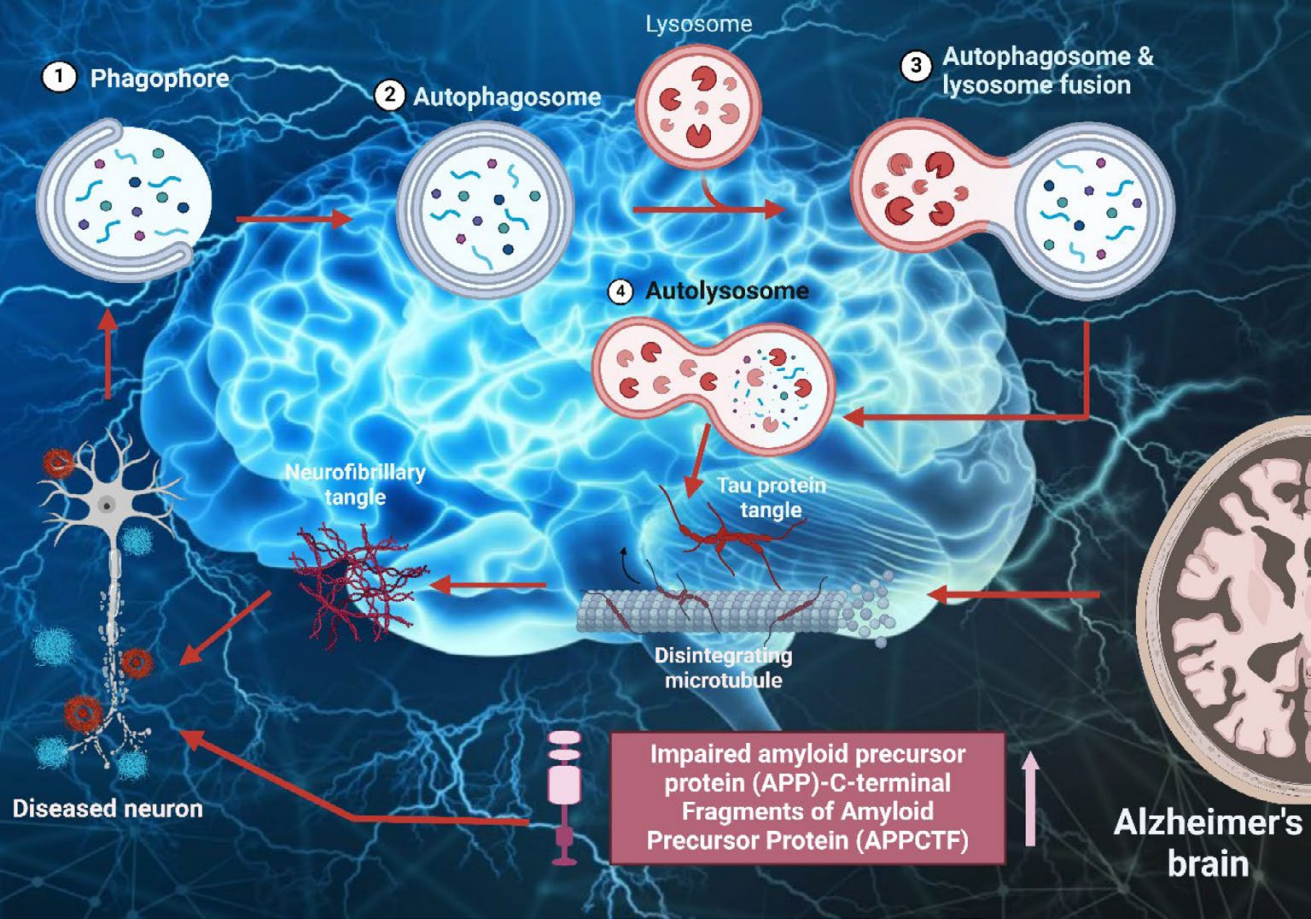
Interactions between signaling systems implicated in cellular senescence in aged neurons. During aging, many variables stimulate the DNA damage response (DDR). DDR enables the triggering of p19ARF, NEMO shuttling, and p38MAPK via ATM and ATR. When p19ARF is engaged, it helps to stabilize p53.

Aβ peptides are formed when amyloid precursor protein (APP) is broken down by β‐secretase (β‐sec) and γ‐secretase (γ‐sec) enzymes [52]. The electron microscope investigations conducted on the AD brain revealed the first proof that autophagy serves a significant part in the process of neurodegeneration [53]. AD brains exhibited a high abundance of autophagic vacuoles (AVs), particularly inside dystrophic neuritis. Autophagy was seen in afflicted neurons' cell bodies, particularly those containing NFTs. Large amounts of undeveloped AVs in damaged nerve fibers indicate that AVs' movement and their transformation into lysosomes may be hindered in AD. Later, research using immunolabeling techniques found that AVs in the brain are a significant storage place for intracellular Aβ [54, 55]. Purified AVs consist of both intact APP and APP that have been cleaved by β‐sec. These AVs are also packed with the components of the γ‐sec complex, namely PS1 and nicastrin (Nt). Furthermore, regulating autophagy in brain cells by the mammalian target of rapamycin (mTOR) kinase results in simultaneous alterations in AV development and Aβ generation [56, 57, 58, 59]. The observations indicate a clear connection between β‐amyloidogenic and cell survival systems via the activation of autophagy in AD. Figure 3 represents the autophagy‐lysosome pathway (ALP) impairment in AD.

The first indication that autophagy may participate in the advancement of AD was derived from detecting many AVs in people with AD. The researchers used immunogold labeling and electron microscopy to find a significant buildup of immature AVs in dystrophic neurites [53]. The purified AVs contained APP and β‐cleaved APP. The vacuoles contain a high concentration of PS1 and Nt, along with Aβ. Further, other data indicate that the transportation of AVs and the fusion of autophagosomes with lysosomes may be hindered in AD. The diminished regressive transportation of autophagosomes and the axon in neurons is essential for their fusion with lysosomes. This reduced mobility results in a buildup of immature AVs and AVs that generate Aβ in AD. This formation causes an increase in Aβ fabrication [54, 60]. Furthermore, it has been demonstrated that Aβ may modulate autophagy by stimulating the creation of AVs via the stimulation of AMPK [61]. Additionally, Aβ has been found to activate autophagy by interacting with Beclin (Bcl)‐1, located in lipid rafts [62].

A fascinating paper has emerged, demonstrating the role of PS1 in lysosomes that are separate from γ‐sec. Lee et al. (2010) showed that PS1 alters lysosome functioning and contributes to the loss of neurons despite PS1 being an important part of the APP‐cleaving γ‐sec. Lysosomal malfunction in PS1 null blastocysts leads to the inhibition of substrate proteolysis and autophagosome elimination. This dysfunction is driven by an absence of PS1‐dependent targeting of the v‐ATPase V0a1 subunit to lysosomes [63]. UPS and autophagy participate in the removal of soluble tau in their monomeric and oligomeric forms and insoluble tau complexes. Specifically, the application of chloroquine to prevent the fusion of autophagosomes and lysosomes leads to a delay in the removal of tau proteins and the buildup of tau clumps [64]. Phosphorylated tau (pTau), one of the several types of tau, is impacted by autophagy malfunction [65].

Furthermore, shortened tau is present in individuals with AD 67 brains. It is eliminated by autophagy in the tetracycline‐inducible model, whereas the proteasome breaks down full‐length tau [66]. Additionally, a reduction of Bcl‐1 has been seen in early‐onset AD (EOAD) and is a result of caspase cleavage [67, 68]. The loss of Bcl‐1 hampers the process of retromer transport and phagocytosis in microglia [69]. It also has an impact on APP metabolism in neurons [70]. Additionally, the decrease in Bcl‐1 leads to a rise in the accumulation of Aβ and neurodegeneration in APP transgenic mice, which serve as an AD model [67]. Research indicates that autophagy participates in the buildup of Aβ and the development of NFTs.

Since aberrant aggregate formation is the primary cause of most NDs, uncovering innovative methods to improve the breakdown of harmful protein complexes is crucial. This is necessary to develop effective treatments for these illnesses. Autophagy is seen as a promising therapeutic focus—furthermore, autophagy safeguards against many stressors and apoptotic damage [71]. Therefore, discovering substances promoting autophagy may be an effective treatment approach. Researchers have been investigating non‐toxic small molecules (SMs) that may restore the expression of NAg. Autophagy‐promoting drugs have significant medicinal uses in several animal models of NDs. Rapamycin improves Aβ and tau's condition in an animal AD model [72]. Latrepirdine, often called dimebon, induces Atg5‐dependent autophagy in the mouse brain and decreases Aβ [73]. The compound metformin, which activates protein phosphatase 2A (PP2A), is being investigated in clinical studies for its ability to prevent the excessive phosphorylation of tau. This effect is achieved by reducing the activity of TORC1 [74, 75]. Furthermore, SMER28, an SM that enhances the activity of mTOR, significantly reduces the amount of Aβ peptide and APP‐β‐C‐terminal Fragment (β‐CTF) in a way that is not reliant on γ‐sec [76].

Autophagy may also be triggered by stimulating the ULK1 kinase, AMPK, in a way that is not reliant on TORC1. Recent lab and patient studies have shown that lithium might improve AD development by activating AMPK and regulating autophagy [77]. Resveratrol, along with its analogs RSVA314 and RSVA405, has many molecular activities, including the stimulation of AMPK. These compounds have been shown to have advantageous benefits against AD [78]. Nicotinamide inhibits the development of disease and cognitive decline by improving the acidity of lysosomes and autophagosomes, reducing the buildup of autophagosomes in a mouse model of AD [79]. Although there is not enough pathological evidence in AD, using virally packaged Bcl‐1 and SMs Bcl‐1 mimetics may also reduce the buildup of harmful clumps by focusing on the first phase of autophagy [80]. By breaking down these clusters, autophagy‐promoting drugs can potentially avoid the buildup of plaques and tangles in the EOAD. Nevertheless, the activation of autophagy may worsen the pathologies in the later phases of AD, especially when there is a dysfunction in the fusion of autophagosomes/lysosomes or the functioning of lysosomes [81]. Hence, developing novel techniques to improve the fusion of autophagosome/lysosome and promote lysosomal functioning would be crucial for advancing AD treatment.

### 3.1 Autophagy in Aβ Metabolism

Autophagy has a role in the metabolism of Aβ, most likely via regulating its generation and removal. Aβ is derived by the enzymatic breakdown of its progenitor protein APP. This breakdown occurs via a sequential cleavage process involving β‐sec (BACE1) and γ‐sec [82]. Cavieres et al. have shown that a certain chemical triggers autophagy, which relies on the ATG5 protein, and this process leads to the increased breakdown of APP [83]. Furthermore, it was revealed that both APP and the four members of the γ‐sec are present inside autophagosomes. This indicates that the autophagic route generates at least several Aβ [84].

Furthermore, it has been shown that autophagy is necessary for generating Aβ and its release. ATG7 functions as the E1‐like enzyme in the process of Ub‐like conjugation during autophagosome generation. Nilsson et al. created a mouse strain called ATG7 KO and bred it with an animal model of AD. The research demonstrates a substantial decline in the production of extracellular Aβ plaques in AD mice with a deficiency in autophagy due to the knockout (KO) of ATG7.

Nevertheless, there was a significant buildup of intraneuronal Aβ, suggesting that the secretion of Aβ was hindered due to defective autophagy [85, 86]. Specifically, recent clinical research found that individuals with deleterious ATG7 mutations have diverse neurodevelopmental problems. The variations result in a substantial decrease or total absence of ATG7 protein, leading to significant autophagy impairment. Collier et al. (2021) found that individuals under 30 with two variants of a particular gene had decreased learning capacity [87]. This suggests a possible link between malfunctioning autophagy and cognitive impairment.

Autophagy has been shown to regulate the removal of Aβ. Earlier investigations have shown that the administration of rapamycin resulted in an augmentation of autophagy in AD model mice. The study found a substantial decrease in intracellular and extracellular Aβ accumulation, leading to better animal mental functioning [72, 88]. Microglial endocytosis is thought to be responsible for eliminating extracellular Aβ [89, 90, 91]. Furthermore, reliable outcomes were achieved in animal models that were manipulated genetically, along with the use of chemical substances. For instance, in mice, introducing a specific mutation (F121A) into the gene that codes for Bcl‐1 disrupts the connection between Bcl‐1 and Bcl‐2, increasing the activation of basic autophagy. Rocchi et al. exhibit that the Bcl‐1 F121A mutation in the AD model leads to a decline in Aβ and averted cognitive impairment [92]. According to this research, in mice with AD with a gene called Bcl‐1 KO, there was an accumulation of intraneuronal and extracellular Aβ compared to the animals in the control group [67]. Autophagy seems to impact the removal of Aβ at various points in the process. Cathepsin (Ctp) B is an essential lysosomal enzyme that breaks down autophagic substances. Studies have shown that removing the gene responsible for Ctp B resulted in a deterioration of AD symptoms in mouse models. This included a spike in the amount of Aβ42 protein and the accumulation of Aβ. In contrast, the overexpression of Ctp B by lentiviral transduction decreased Aβ, even in older animals with AD [93]. The findings indicate that promoting autophagic flow, regardless of the stage, is advantageous in reducing the course of AD.

Selective autophagy has a role in Aβ breakdown, besides its role in bulk autophagy. The process of selectively degrading protein aggregates is called aggrephagy [94]. Earlier studies have discovered numerous distinct autophagy receptors (AgR) involved in aggrephagy, including p62/SQSTM1, NBR1, and OPTN [95]. Typically, Aβ molecules join with Ub to create an association that is resistant to forming clumps of insoluble fibrils [96]. The complex is likely subject to disintegration via the UPS [97]. If Aβ undergoes polyubiquitination, it will probably form insoluble fibrils [98], which are not easily broken down by UPS‐mediated destruction [99]. Alternatively, the fibrils would likely be identified by AgR and broken down by aggrephagy [95, 98, 100].

### 3.2 Autophagy in Tauopathies

Autophagy is also crucial in developing tau disease and its involvement in Aβ degradation. Initially, experiments conducted outside of a living organism showed that the ability to remove tau protein was impaired when autophagic flux was hindered. There was a notable increase in the buildup of insoluble tau complexes [64]. Researchers have discovered that in the central nervous system (CNS) of individuals with familial AD, there is a presence of hTau, which is detected along with the autophagosome indicator LC3 and the AgR p62/SQSTM1. This association was not identified in control participants who do not have NDs [101]. LC3 and p62/SQSTM1 immunoreactivity was repeatedly seen in tau clumps in a cell line representing tau pathology [102]. These results suggest that autophagy also affects tauopathies. Phosphorylation of tau was significantly increased in ATG7 conditional KO (cKO) mice, probably due to the buildup of GSK3β, which participates in tau phosphorylation, in these mice [103].

Autophagy induction has been indicated to ameliorate tauopathies following its involvement in Aβ degradation. The mouse model, which has a mutant form of tau, underwent therapy with mTOR. This treatment resulted in a decline in the levels of pTau. On the other hand, animals with a KO of TSC2 (a protein that inhibits mTOR) had a constantly active mTOR system, which led to increased amounts of tau and its phosphorylation [104]. Recent research discovered many mTOR antagonists that have more potency than mTOR. These drugs were then employed to treat neurons derived from human neural progenitor cells (NPCs) with the tau variant. The findings demonstrated a decline in tau phosphorylation and insoluble tau due to the discovered chemicals [105]. This further supports the notion that accelerated autophagy improves tauopathies. Other molecules in the autophagic pathway also target tau‐related disease and mTOR. Kim et al. have noticed that the AgR NDP52 and p62/SQSTM1 may identify pTau in AD mice [106]. Chesser et al. observed that the increase in NDP52, caused by a chemical found in tea extract, increased the removal of pTau by NAg [107]. In addition, the upregulation of NDP52 via its upstream transcription factor Nrf2 has been demonstrated to enhance the clearance of pTau [95, 108]. TFEB, a key modulator in autophagy, is considered a crucial element in tau diseases, along with mTOR and AgR. Numerous investigations have shown that increasing the activity of TFEB in mice with tau protein abnormalities significantly decreases the levels of pTau, which are soluble and insoluble tau aggregates.

Additionally, there was an improvement in cognitive skills [109, 110]. Recent research suggests that TFEB facilitates the removal of tau by regulating the release of tau from lysosomes. The cKO of TFEB in tau P301S transgenic mice reduces the amount of tau in the interstitial fluid. Still, there is an elevation in tau phosphorylation inside neurons and an enhancement in the dissemination of tau [111].

Tau has two motifs in its C‐terminus that may be identified as a substrate for CMA. However, tau variants are directed to lysosomes by LAMP2A oligomerization but cannot be fully delivered into the lumen [112, 113]. Yet, the introduction of mutated tau into the lysosomal lumen is halted before completion. After the degradation of the lumen portions, the smaller pieces left oligomerize on the lysosome membrane and obstruct the CMA 114–116 process. Research has shown that CMA does not easily break down tau acetylation and mutant tau and that inhibiting CMA in a mouse model worsens tauopathies [114]. Simultaneously, the researchers discovered that the absence of CMA speeds up the tau aggregation process and contributes to the advancement of the illness. Conversely, increasing CMA functioning with SMs reduces AD's severity in two distinct mice models [115]. This data suggests that macroautophagy and CMA are crucial in regulating tauopathies.

### 3.3 Autophagy in Synaptic Function

Synapses are specialized structures in neurons that facilitate communication between presynaptic and postsynaptic neurons. A significant reduction in synaptic density has been observed during the EOAD [116], underscoring the importance of synaptic health in cognitive function. Functional autophagy is essential for maintaining synaptic integrity by regulating neurotransmission, synaptic plasticity, and receptor turnover [117].

Evidence suggests that autophagy participates in the degradation of synaptic vesicle components and neurotransmitter receptors such as GABAA and AMPA receptors, which are critical for synaptic transmission [117, 118]. Disruption of autophagy can impair these processes, contributing to synaptic dysfunction—an early hallmark of AD.

Synaptic plasticity, particularly long‐term potentiation (LTP) and long‐term depression (LTD), underlies learning and memory. Studies have shown that dysregulation of autophagy affects these processes. For instance, a shortage of brain‐derived neurotrophic factor (BDNF) increases autophagosomal accumulation and compromises LTP [119]. Interestingly, while suppression of autophagy has been reported to restore LTP under certain conditions, other studies demonstrate that autophagy activation in the hippocampus is necessary for memory formation, suggesting a context‐dependent role [120]. These findings highlight that precise regulation of autophagy is critical for maintaining synaptic health, and its dysregulation may contribute to the early cognitive deficits observed in AD.

### 3.4 Autophagy in Mitochondrial Dysfunction

Mitochondria serve as the organelles responsible for energy generation. Neurons rely on functional mitochondria to regulate calcium levels, support synaptic plasticity, and ensure the longevity of cells [121]. Reactive oxygen species (ROS) are created as by‐products during energy creation. The buildup of ROS is harmful to mitochondria. Several quality control mechanisms have been found to preserve mitochondrial health and balance [122, 123]. Aβ buildup generates an excessive amount of ROS and results in significant destruction of mitochondria [124]. Mitophagy is the primary method to remove dysfunctional mitochondria [125, 126, 127]. In mammals, mitophagy is often stimulated by the decline of mitochondrial membrane potential (MMP) due to an excessive accumulation of ROS, except in rare instances, including sperm mitochondria in fertilized eggs and developing red blood cells (RBCs) [128]. The PINK1‐Parkin system (Prk) is a well‐investigated route that regulates MMP‐dependent mitophagy [129]. PINK1 is a serine/threonine protein kinase found in the cytoplasm. In normally functioning mitochondria, it is transported into the mitochondrial matrix. When mitochondria become infected, the MMP gets impacted and prevents the import of PINK1. As a result, PINK1 remains on the outer MMP (OMMP), where it becomes active by autophosphorylation [130, 131, 132]. PINK1 becomes active and phosphorylates Prk and Ub on the OMMP, producing phospho‐Ub [133, 134]. Prk, functioning as an E3 ligase, identifies the phospho‐Ub on the OMMP, attracting more Prk [135]. Ubiquitinated mitochondria are often determined by p62/SQSTM1 and OPTN and undergo autophagic destruction [129, 136]. Alternatively, it has been shown that Prk is not necessary and that phosphorylated Ub by PINK1 alone is powerful enough to identify NDP52 and OPTN [137]. Research has discovered PTEN‐L, a new type of PTEN, as a crucial inhibitor of PINK1‐Prk‐driven mitophagy. PTEN‐L inhibits mitophagy by dephosphorylating Prk and Ub via its inherent phosphatase function [138]. Aside from PINK1‐Prk‐mediated mitophagy, many additional types of mitophagy have been documented [122, 126, 129], highlighting the intricate nature of maintaining mitochondrial stability.

AD is often associated with malfunctioning mitochondria. Postmortem examinations have shown that the process of removing damaged mitochondria (mitophagy) in the hippocampus was significantly decreased in individuals with AD. Additionally, as mitochondrial damage is an initial clinical characteristic of AD and the entorhinal cortex is the first area to be impacted by AD, it has been suggested that impaired mitophagy in the entorhinal cortex might be an EOAD pathology [139]. The AD mice models and neurons produced from induced pluripotent stem cells (iPSCs) of persons afflicted by AD had comparable behavior, indicating that stimulating mitophagy might potentially improve the progression of AD [140, 141, 142]. The research used pharmacological agonists of mitophagy to cure cells and organisms with AD. The findings indicated that increased mitophagy reduced the presence of both Aβ and tau and benefited the cognitive functioning of AD *Caenorhabditis elegans* and mice models [140]. While more investigation is required to comprehend the connection between mitophagy and the development of AD, the process of mitophagy, which involves removing damaged mitochondria, shows promise as a possible therapy for treating AD.

## 4 Cellular Senescence in the Aging Brain

**FIGURE 4:**
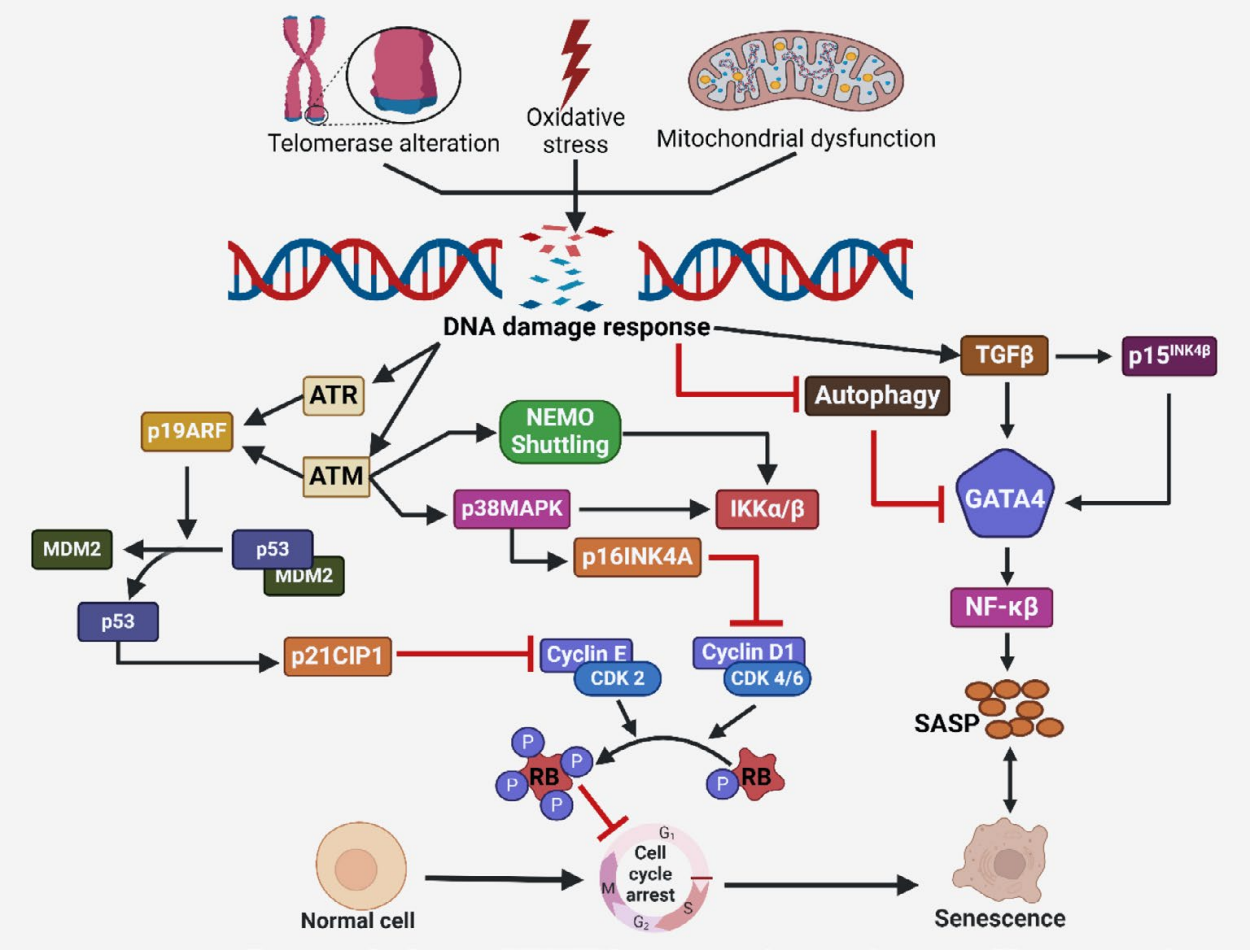
Interactions between signaling systems implicated in cellular senescence in aged neurons. During aging, many variables stimulate the DNA damage response (DDR). DDR enables the triggering of p19ARF, NEMO shuttling, and p38MAPK via ATM and ATR. When p19ARF is engaged, it helps to stabilize p53.

While cellular senescence and aging are often discussed concerning each other, it is crucial to acknowledge that these processes are distinct from one another and do not necessarily happen simultaneously, as emphasized by experts [143]. The question of aging and its causes, duration, and biological importance continues to be a matter of vigorous discussion among scholars and investigators. Aging is characterized by the gradual decline of biological functioning, reduced capacity to reproduce, higher likelihood of ARDs, and increased death as time passes [144, 145]. Nevertheless, there is a contention that this description is just for creatures found in their natural habitats [146]. In the last 50 years, researchers have suggested numerous proposals to elucidate the mechanisms of aging at the cellular and molecular stages. According to current understanding, aging is often recognized as a pseudo‐programmed occurrence that is caused by random causes instead of being a deliberate reaction that has been there from the beginning of humanity [147]. Aging at the cellular level is caused by random changes, such as variations, injury to DNA, and the buildup of free radicals. These changes then activate planned processes that oppose them, such as metabolic adaptation, DNA damage response (DDR), neutralization of ROS, cellular senescence, or programmed cell death (PCD). As one ages, their capacity to counteract random harms decreases, leading to a deterioration in function, increased vulnerability to infections, and finally, death. However, senescence is a biological reaction to stress signals that are caused by the process of aging. Senescence primarily restricts the development of aging‐induced damaged cells by enforcing a persistent cessation of growth. Therefore, senescence prevents the advancement of cancers and the transmission of possibly altered genetic material to future generations [148, 149]. SCs involve a series of steps, including stopping the cell cycle, activating DDR, increasing metabolism, and improving resistance to cell death. These processes may have negative impacts on the body later in life [147]. From a molecular perspective, aging and cellular senescence are influenced by a shared group of factors, including genetic instability, telomere shortening, damaged mitochondria, and pro‐inflammatory cytokines (ProICs) [148]. The functional relationship between cellular senescence and aging implies that both play an intertwined role in organismal aging. However, they seem to be controlled separately in various respects. Figure 4 illustrates a diagrammatic summary of the interrelated chain of signaling channels that serve in cellular senescence in aged brain cells.

On the other hand, NEMO shuttling and p38MAPK initiate the NF‐κB system. DDR also facilitates GATA4 via TGF‐β stimulation and inhibits autophagy, resulting in the stimulation of NF‐κB. NF‐κB, when triggered, plays a role in actively releasing SASP components and also helps in facilitating cell cycle arrest. DDR also induces the stimulation of p16 concurrently. The activation of p16 and p21 prevents the cyclin‐CDK complex from suppressing the hyperphosphorylation of pRB, hence blocking the advancement of the cell cycle—cellular senescence results from the combined effect of SASP and cell cycle arrest.

Ataxia Telangiectasia Mutated (ATM), Ataxia Telangiectasia and Rad3‐related protein (ATR), GATA Binding Protein 4 (GATA4), Murine double minute 2 homolog (MDM2), nuclear factor‐kappa B (NF‐κB), nuclear factor erythroid 2–related factor 2 (NRF2), p38/mitogen‐activated protein kinase (p38/MAPK), SASP, and transforming growth factor‐beta (TGF‐β).

SCs secrete various molecules linked with the SASP, such as ProICs, chemokines, matrix metalloproteinases (MMPs), and ROS [21, 150]. These SASPs spread to adjacent cells, resulting in paracrine senescence of the adjacent cellular milieu. The triggering of SASP stimulates many signaling systems, including MAPK, DDR, p38, and mTOR networks. Geroconversion occurs when these channels are activated, leading to a transition from reversible to irreversible cell cycle halting, a significant characteristic of senescence. At the level of the organ, geroconversion leads to an increase in size and a condition of excessive secretion, marked by inflammation and dysfunction. This condition increases the likelihood of the aging organ developing ARDs [147, 151]. Therefore, it may be inferred that senescence is a catalyst for the aging process, yet it is also plausible that senescence is an outcome of aging.

The hippocampus is a well‐researched area in the study of aging, primarily because it is essential in memory and cognition, both of which are affected by aging. The decrease in hippocampus thickness due to aging has been associated with the deterioration in cognition, as shown in multiple investigations [152]. The role of SCs in aging is well recognized. Baker and his colleagues have researched this area significantly [153, 154]. While cellular senescence plays a role in suppressing tumors, it may also stimulate the synthesis of SASP components [21, 155]. Mice with mutated genes resulting in decreased levels of the DNA repair endonuclease enzyme ERCC1‐XPF showed increased mRNA levels of p21CIP1 and p16INK4A compared to normal‐aged mice. The alteration also increased SASP variables like IL‐1β, IL‐6, tumor necrosis factor α (TNFα), monocyte chemoattractant protein‐1 (MCP‐1; CCL2), and others in several organs, including the brain, kidney, liver, and spleen [156]. Although research acknowledges the participation of SCs in aging, their relevance remains a subject of ongoing controversy. The investigators have proposed that increasing SCs throughout the aging process could serve as a compensatory strategy to preserve tissue function [157, 158, 159, 160, 161, 162].

### 4.1 Cellular Senescence in Neurons

Neurons are distinct from other brain cells because they do not undergo cell division, making them postmitotic. Mouse‐aged neurons exhibit characteristic signs of senescence [163, 164, 165]. Furthermore, the aging characteristics of neurons are reversed by eliminating CDKN1A in mice, suggesting that p21 is responsible for senescence in older neurons [165]. One further hallmark of mature neurons is the buildup of cytoplasmic deposits, such as lipofuscin, a common feature of non‐dividing aging cells. Lipofuscin consists of intricate combinations of proteins, lipids, and sugars that are extensively cross‐linked. It occurs when elevated quantities of lipid peroxidation products react with lipids, proteins, and other vulnerable groups. The primary factors contributing to the production of lipofuscin are changes in proteostasis and malfunctioning of mitochondria. Lipofuscin concentrations rise throughout aging but appear expedited by pathological factors, including neuronal loss, gliosis, OS, and malfunctions in mitochondria and lysosomes [166]. Lipofuscin is present in the neurons of NDs such as AD. For example, in EOAD, lysosomes become saturated with lipofuscin and develop into a substantial buildup of massive lipofuscin aggregates inside the cytoplasm [167, 168]. Remarkably, lipofuscin is present in the extracellular compartment after neuronal demise and often coexists with Aβ, indicating a potential association between lipofuscin and the development of senile Aβ in AD [167, 168, 169]. α‐syn was detected in lipofuscin accumulations of neurons in the substantia nigra pars compacta (SNpc) in individuals with PD [170]. There is a clear correlation between the timing of lipofuscin production and the accumulation of protein clumps in developing NDs [171, 172]. The relationship between lipofuscin production and protein clumps, such as Aβ, α‐syn, or tau, is not definitively understood. However, research suggests that there could be a connection with SCs.

Finally, a fascinating feature of neural aging is an activated reaction to damaged DNA that prevents cells from re‐entering the cell cycle and undergoing PCD [173]. Substantial DNA damage causes the stimulation of DDRs, which result in the release of ProICs like IL‐6. Additionally, it regulates the p53/p21 systems that participate in DNA repair, cell cycle arrest, and PCD [173, 174]. As a result, neurons that have survived but are showing signs of aging may produce substances that promote inflammation, leading to long‐term immunological responses and ongoing effects on the surroundings that may impact various cells in the CNS.

### 4.2 Cellular Senescence in Astrocytes

Astrocytes have a wide range of functions, including neurogenesis, preserving extracellular homeostasis, and modulating BBB permeability [175]. Eventually, cultured astrocytes exhibit characteristics similar to those seen in replicative senescence due to stress [176, 177, 178]. Astrocytes showed an elevated expression of SA‐β‐gal, generation of ROS, p53, p21CIP1, p16INK4a, and SAHF, as well as the activity of astrogliosis markers like glial fibrillary acidic protein (GFAP) and S100β. It is crucial to note that astrocytes in the aging brain exhibit SASP traits, leading to heightened release of ProICs like IL‐6 [179]. Furthermore, the survival of neurons co‐cultured with senescent astrocytes (SAs) was lower than that of non‐SAs. This suggests that SAs could potentially impact neuronal existence [177]. Neurons co‐cultured with aging astrocytes also exhibited a decline in glutamate release from synapses and reduced synaptic vesicle pools [180]. These specialized cells, including perivascular astrocytes, pericytes, and other endothelial cells (ECs), wrap around cerebral circulation with their broadened end [181]. In animal models, the breakdown of the BBB is linked with astrogliosis through the secretion of IL‐6, MCP‐1, CCL2, and MMP9. Modifications in the extracellular environment can potentially impact interactions among adjacent neurons and other glial cells [182].

### 4.3 Cellular Senescence in Microglia

Microglia show the usual signs of replicative senescence [183]. Microglia from older mice exhibit elevated levels of ProICs, including IL‐6, IL‐1β, and TNFα, which are commonly increased throughout SCs [184]. Senescent microglia (SM) have been observed in various regions of the aging brain [185, 186, 187]. Senescence has an impact on the makeup as well as the functioning of microglia. SM exhibits distinct morphological characteristics compared to reactive microglia, often displaying deramification, beading, and cytoplasmic fragmentation [185]. SM also show decreased movement, which impacts their capacity to move toward the injured area, where immunity is needed [188]. Transcriptome evaluations of aged microglia have revealed an upregulation of specific genes linked to NDs, such as Triggering receptors expressed on Myeloid Cells 2 (TREM2) [189, 190, 191]. Microglia participate in the brain by ensuring iron levels are balanced through the uptake and storage of iron in ferritin proteins [192, 193]. Iron serves a crucial part as a cofactor in mitochondrial respiration, which is essential for the generation of energy [194]. Nevertheless, microglia that have become senescent may experience a decline in their ability to retain iron, resulting in increased levels of iron and OS within the brain.

### 4.4 Cellular Senescence in Oligodendrocytes

Oligodendrocytes (OGDs) are specialized cells that perform an essential function in the CNS by myelinating neuronal axons. These cells are particularly susceptible to OS due to their elevated expression of glycolysis and mitochondrial activity [195, 196]. Through the process of glycolysis, the level of acetyl‐CoA is elevated, which in turn supports the production of fatty acids necessary for myelination. It has been observed that older adults show signs of oxidative DNA damage and raised SA‐β‐gal in their OGDs. This indicates that these cells may experience cellular senescence due to stress [163, 197]. Oligodendrocyte progenitor cells (OPCs) are the predecessors to mature OGDs. These cells migrate toward injured neurons and restore the myelin sheath around axons as part of the healing process. As we grow older, the regenerative capacity of damaged axons tends to decline, which is associated with the senescent characteristics seen in OGDs and OPCs. When studying senescent OPCs in culture, researchers observed a rise in the levels of Ecrg4, a gene associated with esophageal cancer. This gene promotes senescence by speeding up the degradation of cyclin D1 and D3 cell cycle genes through the proteasome‐dependent pathway. Therefore, Ecrg4 effectively suppresses the continued growth of OPCs, decreasing the overall OPC population [198]. Furthermore, senescent OPCs release Ecrg4, which can trigger senescence in neighboring OPCs. These findings indicate that senescent OGDs and OPCs no longer contribute to neurons' regeneration and energy supply.

### 4.5 Comparative Insight Into Other Neurodegenerative Diseases

In addition to AD, autophagy and cellular senescence are implicated in other NDs such as PD and FTD. In PD, dysfunctional mitophagy, particularly involving the PINK1/Parkin pathway, plays a central role in dopaminergic neuronal loss [199], while SAs and microglia contribute to neuroinflammation [200]. FTD, frequently associated with tauopathy and TDP‐43 aggregation, exhibits impaired autophagic flux and accelerated senescence in cortical neurons [201]. Although overlapping mechanisms such as OS and mitochondrial dysfunction exist, AD tends to feature earlier autophagy–lysosomal system impairment, while PD displays more pronounced deficits in mitophagy, and FTD is characterized by region‐specific vulnerability of frontal and temporal cortices [202]. Comparative studies may reveal disease‐specific senescence–autophagy interdependencies that can be leveraged for targeted therapeutic strategies.

## 5 Cellular Senescence and AD

Both preclinical and clinical research prove that cellular senescence is a crucial factor in the progression of several ARDs, including AD [203, 204, 205, 206, 207]. Age is regarded as the primary risk factor for AD, notwithstanding the unclear process behind the increased vulnerability to AD associated with aging. Research conducted on both humans and animals has provided findings that show that cellular senescence is a crucial factor in the progression of several disorders associated with aging, such as AD. Senile plaques, characterized by Aβ outside the cells, and NFTs, are two pathological characteristics of AD [208, 209, 210]. While there is ongoing debate on the exact mechanisms by which Aβ and tau contribute to neurodegeneration, it is widely accepted that both Aβ and tau disorders vigorously promote cellular senescence. SCs have been seen in the brains of individuals with AD [205, 206, 207, 211, 212, 213, 214], as well as in mouse models of AD that have excessive production of Aβ or tau protein [207, 211, 215, 216]. Treating AD model mice with pharmacological and genetic methods effectively decreased the accumulation of SCs in the brain, decreasing Aβ burden and tauopathy. Additionally, this treatment led to an improvement in cognition [207, 211, 215]. The results indicate that cell senescence is vital in developing neuropathophysiology (NPP) caused by Aβ and tauopathy in AD. Furthermore, studies indicate that SCs actively contribute to developing Aβ and tau disorders [205, 217]. Understanding the processes that cause neurons to age and become senescent, both in normal aging and in AD, as well as how these aging cells participate in the deterioration of the brain in AD, will be crucial for developing ways to avoid or reverse this debilitating condition.

### 5.1 Evidence of SCs in AD Patients

While there is a growing body of data indicating neuronal senescence in animal models of AD, there is currently limited proof of cell senescence in the brains of individuals with AD. This is primarily because no precise and effective indicators are available for identifying SCs in living organisms and samples taken after death. However, many investigations utilizing immunostaining methods have demonstrated that various cell types, such as astrocytes, microglia, neurons, and ECs, in the AD brain exhibit elevated concentrations of senescence‐linked proteins, such as cell cycle inhibitors p16, p53, and p21 [205, 207, 213, 218, 219, 220, 221, 222, 223]. This indicates that cellular senescence might contribute to the development of AD.

Naderi et al. found that fibroblasts obtained from persons with AD have higher concentrations of p21 but a lower concentration of Bax, a hallmark of death, compared to those obtained from people without AD [220]. Senescent ECs (SECs) have also been seen in the frontal and temporal cortex of individuals with AD [221]. Turnquist and Bhat demonstrated an upsurge in the quantities of p53β, a coactivator of full‐length p53, and p16‐positive astrocytes in the brains of individuals with AD. In a state of senescence, these astrocytes released several ProICs, including IL‐6, a sign of senescence [205, 218]. In contrast, Zhang et al. found that OPCs in the AD brain had a senescence‐like phenotype. This was marked by elevated levels of p21 and p16 and enhanced functioning of SA‐β‐gal. It is worth noting that astrocytes, microglia, and OGDs did not show these characteristics [211]. The continuous increase in microglia is a characteristic feature of AD. Investigation showed that the rapid and continuous growth of microglia in APP/PS1 mice, a widely used mouse model for familial AD, and in postmortem tissue of individuals with AD led to the development of senescence‐like characteristics. These characteristics included elevated SA‐β‐gal function, telomere reduction, and a transcriptional signature linked to senescence. Inhibition of initial microglial growth impeded the progression of microglial senescence, Aβ buildup, and destruction of neurites and synapses in APP/PS1 animals [219]. According to their findings, continuous cell division may cause microglia in the AD brain to undergo replicative senescence.

One of the characteristic features of the brain affected by AD is the presence of NFTs formed by Tau proteins. In their study, Musi et al. used laser capture microdissection methods to demonstrate that neurons containing NFTs, rather than the neurons bordering Aβ plaques, exhibited a senescence‐like phenotype in both tau transgenic mice and brain tissue affected by AD [207]. Gaikwad et al. demonstrated that astrocytes in the AD brain had a senescent character when they were near pathogenic tau oligomers [222]. Endothelial dysfunction, particularly vascular dysfunction, significantly contributes to the development and advancement of AD. Bryant et al. conducted a comparison of the activity of 42 genes related to cellular senescence and adhesion in preserved microvessels extracted from the dorsolateral prefrontal cortex of 16 persons with advanced AD (Braak V/VI, B3) and 12 healthy individuals [224]. Researchers discovered that multiple proteins linked to cellular senescence and adhesion, such as Serpine1 (also known as PAI‐1), a marker for cell senescence and a serine protease inhibitor, were notably elevated in B3 microvessels of AD individuals compared to the control group. These findings were adjusted for sex and cerebrovascular pathology [224, 225, 226, 227, 228, 229, 230, 231]. According to their findings, it is indicated that ECs in individuals with AD may experience senescence.

A telomere is a segment of DNA consisting of repeated sequences of nucleotides located at the terminal end of a chromosome. Its primary function is to safeguard the chromosome against degradation. Insufficient DNA replication throughout the cell cycle deletes a portion of the telomere, causing chromosomal instability. Replicative senescence results from telomere shortening, causing a permanent halt in the cell cycle. Several investigations suggest a correlation between telomere shortening and the aging process and age‐related disorders, such as AD [232, 233, 234, 235, 236, 237, 238]. However, reports have also indicated no relationship between the two [239, 240]. Lee et al. found that the frequency of telomere shrinkage per year in peripheral leukocyte DNA is higher in patients with AD than those in good health and those with moderate cognitive decline [237]. Liu et al. demonstrated a more pronounced inverse relationship between telomere length and age in individuals with AD, particularly in females, compared to the controls [241]. Notably, studies have demonstrated an important distinction in telomere length between individuals with AD with homozygous ApoEɛ4 allele and those with heterozygote ApoEɛ4. However, there is a slight variation in telomere length between AD victims and non‐dementia elderly individuals in general [242]. Guo et al. demonstrated a causal relationship between reduced telomere length and an increased risk of AD using the genetic risk score and Mendelian Randomization (MR) approaches [234]. Research has demonstrated a connection between telomere shortening and a quick deterioration in cognitive function, as well as the development of dementia in people with mild cognitive impairment (MCI) [238]. However, there have also been reports of studies that show no connection. Moverare‐Skrtic et al. found that the length of the telomeres in leukocytes decreased in individuals with stable MCI. However, they did not see a correlation between telomere shortening and the development of AD [239]. Hinterberger et al. conducted longitudinal community‐based age‐cohort research and found a connection between leukocyte telomere length and vascular illnesses, but not AD [240].

### 5.2 Evidence of SCs in AD Mice Models

AD is characterized by the excessive buildup of Aβ outside of cells and the buildup of tau proteins within cells. These are two key indicators of the illness [208, 209, 210]. While the reasons for the greater buildup of Aβ and tau proteins in the brains of people with late‐onset AD (LOAD) are not yet understood, researchers have created multiple animal models to investigate the processes by which these proteins contribute to AD development. These models involve the overexpression of a mutated human APP, both APP and mutant human PS1, or a mutant form of human MAPT (P301S). These models are being employed to investigate the processes through which Aβ and tau proteins foster the pathophysiology of AD [207, 211, 215, 216, 222, 243, 244, 245, 246, 247, 248, 249]. Recent findings from these animal experiments suggest that elevated levels of Aβ and tau phosphorylation contribute to the aging of brain cells and that cellular senescence is a crucial factor in developing neuropathology and memory deterioration in AD.

Multiple mouse or rat models have been created to increase the expression of human APP or APP together with mutant human PS1. These models are used to investigate the impact of the buildup of Aβ on the NPP of AD. APP/PS1 mice are genetically modified to express a chimeric mouse/human APP (Mo/HuAPP695swe) and a mutant human PS1 (PS1‐dE9). These mice exhibit a substantial rise in brain Aβ accumulation and cognitive decline, making them frequently employed as a rodent model for familial AD. Recent research indicates that many kinds of neural cells, such as OPCs, astrocytes, microglia, and neurons, exhibit increased expression of senescence markers p16, p21, and SA‐β‐gal in familial AD model mice [211, 216, 243, 244]. Nicotinamide adenine dinucleotide (NAD+) is a crucial molecule that plays a significant role in several biological functions. Hou et al. discovered that decreased NAD+ levels in neurons led to senescence characteristics in several kinds of neurons in APP/PS1 mice. These characteristics included heightened SA‐β‐gal function and elevated levels of p16 and p21. The administration of nicotinamide riboside (NR), a precursor of NAD+, resulted in an elevation in NAD+ levels in the CNS and a decrease in the number of SCs and neuroinflammation. This indicates that the documented cellular senescence and neuropathological alterations in the brains of APP/PS1 mice may be caused by a deficit of NAD+ [244]. The connection between cellular senescence and NPP in APP/PS1 mice has been thoroughly explained in a recent study. Zhang et al. found that AD individuals and APP/PS1 mice, OPCs expressing Olig2 and NG2, which have been linked with Aβ plaques, demonstrate a senescence‐like phenotype. Higher levels of SA‐β‐gal and elevated levels of p16 and p21 mark this phenotype. It is important to note that astrocytes, microglia, and OGDs did not show these characteristics. Exposing produced OPCs directly to accumulated Aβ caused senescence in these cells. Crucially, the researchers demonstrated that administering senolytic medicines to APP/PS1 mice eliminated SCs from the plaque area, decreased neuroinflammation and Aβ accumulation, and improved memory impairments [211]. Their observations indicate that cellular senescence is involved in developing Aβ‐induced neuropathology and memory impairment. Additionally, their findings indicate that cell senescence is an aspect of the buildup of Aβ in individuals with AD.

Various mouse models harboring either normal or mutated human tau protein have been employed to investigate the process of tauopathy participating in the NPP of AD [207, 215, 222, 246, 247, 248, 249]. One of the animal models used is MAPTP301SPS19 (PS19) mice. These mice exhibit the P301S mutant version of the human MAPT under the control of the mouse prion protein promoter. The mice exhibit elevated amounts of mutated human tau, particularly in neurons [248], resulting in gliosis, buildup of NFTs, neurodegeneration, and cognitive decline [215]. Bussian et al. observed elevated expression of the senescence indicator p16 in astrocytes and microglia inside the brains of PS19 animals. Furthermore, they demonstrated that gliosis, tau hyperphosphorylation, and deterioration of cortical and hippocampal neurons were averted by either pharmacologically administering senolytic drugs or genetically clearing p16‐expressing SCs by crossing PS19 mice with INK–ATTAC transgenic mice, which was linked to maintaining cognitive function [215]. The data provide a definitive causal connection between tauopathies, cellular senescence, neurodegeneration, and memory loss.

The forebrain of rTg4510 mice contains mutant human tauP301L. The mice in this study exhibit the development of tau disease in specific areas of the forebrain, which occurs along with neurodegeneration and cognitive impairments [247]. In their study, Musi et al. demonstrated that neurons containing NFTs, but not those connected to Aβ, had characteristics similar to cellular senescence. These neurons also expressed genes related to senescence, such as Cdkn2a, which was directly linked to brain shrinkage and the accumulation of NFTs. Significantly, the researchers demonstrated that administering senolytic medications dasatinib and quercetin (D + Q) to these animals decreased the overall density of NFTs, neuron loss, and swelling of the ventricles. This indicates that cellular senescence plays a role in the loss of neurons caused by tauopathy [207].

NFTs accumulate in AD even when no tau mutations are present. To investigate the development of excessive production of normal tau protein, researchers have created hTau mice. These animals produce six variations of the human MAPT protein but do not produce the mouse tau protein [246]. These animals exhibit age‐related AD‐like characteristics, such as the buildup of hTau in the form of aggregate coupled helical filaments in the cell bodies and dendrites of neurons, neuroinflammation, impaired synapses, and memory impairment [222, 246, 250]. Significantly, these animals have elevated levels of SCs in the brain. Administering high mobility group box 1 (HMGB1) antagonists to hTau animals, which caused astrocyte senescence in vitro due to tau protein, resulted in a substantial reduction in the number of SCs in the brain. The intervention also lowered neuroinflammation and improved cognitive functioning [222]. This result proves that tau protein contributes to memory loss, at least partially, by triggering cellular senescence in the brain.

## 6 Interplay Between Impaired Autophagy and Senescence in AD

Autophagy is a crucial process of lysosome‐mediated degradation that is linked with SCs. The decline in autophagic behavior that occurs with aging is thought to participate in the buildup of impaired proteins and organelles [251]. This reduction in autophagy has been associated with the development of ARDs. Furthermore, growing data indicate that the age‐related decline in autophagy is especially associated with neuronal senescence [252]. Consistently, the inhibition of autophagy during cellular senescence has been seen in cultured neurons [253, 254]. Replicative senescence is expected to occur naturally in dividing cells in the aging brain. Cellular senescence indicators have been identified in certain kinds of cells in the setting of aging and neurological disorders [205].

### 6.1 Autophagy and Lysosomal Dysfunction in AD

Ample proof from the hippocampi of individuals with AD demonstrated increased activity of mTOR and its components before it, indicating a persistent suppression of autophagy [255]. Eliminating mTOR by genetic means in Tg2576 mice stimulated autophagy and successfully reversed memory impairments [256].

AD is characterized by the degeneration of neurons and the formation of AVs in neuronal operations, including synaptic areas [53]. AVs have been seen in people with AD and in mice that have been genetically modified to harbor the mutated P301L Tau. These AVs are believed to represent residues of autophagy that occur in the later stages, indicating a dysfunction in the autophagic pathway during the lysosomal degradation phase [257]. In addition, the Bcl‐1 levels decrease in people with AD compared to healthy individuals [67]. Furthermore, the synthesis of PI3P, facilitated by the Bcl‐1/VSP34 complex, was reduced in individuals with AD [258]. Deposition of AV occurs in the EOAD, even before the buildup of extracellular Aβ plaques. This suggests that the stimulation of macroautophagy is an early reaction in the progression of the illness [54]. AVs purified were concentrated with APP and processed by enzymes, including γ‐sec, responsible for the initial generation and buildup of intracellular Aβ1‐42 in neurons [259]. Autophagolysosomes' neuronal development and retrograde movement are hindered in AD, leading to a notable shortage of lysosomes in normal aging and AD brains [51]. The study demonstrated that interfering with the breakdown of proteins in lysosomes in normal mice replicated the neurological abnormalities observed in AD and worsened the dysfunction of autophagy and the buildup of Aβ in animal models of AD [260].

γ‐sec, in collaboration with β‐sec, cleaves APP to generate the Aβ1‐42 peptide, which is the main constituent of Aβ plaques. The subunits include PS1, PS2, Nt, and APH1. PS1 is a transmembrane protein that acts as a catalyst for γ‐sec and has a role in various physiological processes, such as neuronal plasticity, neurite development, cell binding, and calcium equilibrium, among numerous others [261]. The presence of exacerbated Aβ in individuals with EOAD, caused by abnormalities in the PS1 gene, reveals the involvement of PS1 in the operation of lysosomes [54, 262]. The loss of PS1 inhibits the metabolism of proteins through macroautophagy by disrupting the acidification of lysosomes. This disruption is mediated by the failure of the proton translocating V0a1 subunit of the vacuolar (H+)‐ATPase to mature properly [63].

Furthermore, studies have demonstrated that removing PS1 leads to a reduction in p62, a protein responsible for the breakdown of Tau. This reduction in p62 ultimately hinders the turnover of Tau, resulting in dysfunction [263]. PS1 insufficiency leads to the constant activation of MTORC1, suppressing TFEB‐mediated autophagy and lysosome formation [264]. Furthermore, it has been found that the PS2 mutant hinders the fusing of autophagosomes and lysosomes, affecting autophagic flow [265]. Lysosomal impairment has also been observed in connection with the APP‐β‐CTF in individuals with Down syndrome, which typically progresses to AD [266]. Comprehensive research on CA1 pyramidal neurons in the hippocampus of individuals with EOAD and LOAD has shown that autophagy movement is increased in AD. However, it becomes gradually hindered due to inadequate removal of substances, as exhibited by the buildup of LC3‐II and SQSTM1/p62 in autolysosomes and the enlargement of autolysosomal size and total area [267].

In AD, the malfunctioning of lysosomes leads to the buildup of Aβ1‐42 and abnormal Tau proteins [51]. Transcription factor EB (TFEB) is an essential protein in creating lysosomes. TFEB is typically confined to the cytoplasm through phosphorylation. However, in response to certain stimuli, it moves to the nucleus. It attaches to a particular segment of DNA known as coordinated lysosomal expression and regulation (CLEAR) in the boosters of various lysosomal and autophagic genes, such as WIPI1, PSEN2, LAMP1, CTSB, CTSD, among others [268]. Studies have demonstrated that overexpression and stimulation of TFEB may have the potential to reverse the pathology associated with AD. A fresh investigation found that levels of the TFEB vary depending on the Braak stage, as stated in the reference. The concentrations of TFEB in the nucleus significantly dropped throughout the final phases of the condition. This loss was offset by a rise in its levels in the cytoplasm. This discovery supports the well‐documented observation of lysosomal abnormalities in AD [269]. In the final phases of AD, there is an upsurge in the activation of autophagy, which results in the buildup of Aβ and Tau. As a result, compensatory overexpression of TFEB occurs, which may help in clearing some of the stored Aβ, but it is not enough to eliminate all the stored waste. Amplification or stimulation of TFEB facilitated the removal of Aβ by regulating the ALP, resulting in a decrease in Aβ‐induced generation of ROS and cell death [270, 271, 272]. Nevertheless, these methods seem to be effective in the EOAD, but they are inadequate in the final phases, suggesting the existence of a more impaired autophagic system. TFEB‐mediated autophagic lysosomal pathway (ALP) has been demonstrated to regulate Aβ turnover in neurons under normal circumstances. TFEB enhances APP cleavage by upregulating the α‐sec ADAM10, leading to an elevation in α‐CTF and preventing the formation of Aβ. Furthermore, TFEB potentially increased β‐CTF levels by modifying the process of proteasome‐mediated catabolism [273]. Multiple investigations have indicated that β‐CTF plays a cytotoxic influence [274]. Therefore, the potential of enhancing the lysosomal pathway as a means of therapy ought to be thoroughly assessed. TFEB is likely implicated in quality of life and its role in memory retention. Decreasing the expression of hlh‐30 (the equivalent of the mammalian TFEB) and daf‐16 (the mammalian FOXO) in *C. elegans* resulted in a shorter lifespan for both the normal and long‐lived daf‐2 mutant [275]. Conversely, increasing their expression promoted autophagy and increased lifespan [276].

Increasing the expression of TFEB in both microglia and astrocytes enhances their ability to eliminate Aβ plaques [277, 278]. Overexpressing TFEB in various AD mouse models has been linked to increased removal of NFTs through the ALP without impacting normal Tau levels. This TFEB upregulation also leads to enhanced neuron preservation and synaptic activity. The rescuing of Tau accumulation by TFEB is most likely achieved through the TFEB‐PTEN‐PI3K‐Akt–mTOR system, resulting in elevated loss of Tau by CtpD [109, 110]. It is worth noting that while CtpD polymorphism is a risk variable for AD, a total absence of CtpD is linked with severe NDs [279, 280]. Astroglial TFEB upregulation has been found to boost the lysosomal route and inhibit Tau accumulation in several AD mice models, such as rTg4510 and PS19, in reaction to trans‐synaptic spreading of Tau [281, 282]. TFEB also governs lysosomal exocytosis, which could play an undiscovered function in removing cellular waste in NDs [283]. TFEB plays a role in the process of releasing Tau by exocytosis. TFEB uniquely identifies the microtubule‐binding repeat (MTBR)—shortened Tau, but not the unaltered version—and facilitates its release through the lysosomal calcium channel TRPML1. The absence of TFEB resulted in an increased buildup of Tau [111]. It is worth mentioning that TFEB has shown beneficial effects in several NDs, such as PD and HD, by effectively removing abnormal protein clumps in experimental models [284, 285]. This study indicates that the functioning of lysosomes and autophagy, which TFEB regulates, is essential in maintaining protein homeostasis and protecting the CNS.

### 6.2 Aβ and Tau‐Associated Autophagic Abnormalities

Although the precise chemical connections between Aβ, Tau, and autophagy remain incomplete, new research has revealed their interconnected relationships. Introducing a specific genetic mutation (F121A) in the mouse gene responsible for producing Bcl‐1 disrupted the relationship between Bcl‐1 and Bcl‐2. This disruption ultimately led to the stimulation of autophagy. AD animals with this variant exhibited a significant decrease in Aβ and a restoration of cognition [92]. Bcl‐1 KO mice exhibited intercellular and extracellular Aβ accumulation compared to the control mice [67]. The advantages may be ascribed to the initiation of autophagy, which regulates the elimination and synthesis of Aβ.

Furthermore, the elimination of Bcl‐1, associated with increased AD pathology, along with the targeted reduction of Atg7 in an APP/PS1 mice model of AD, led to an increased release of Aβ, which in turn encourages the creation of extracellular Aβ. Autophagy has been discovered to play an important role in releasing undegraded integral membrane proteins. This release occurs through a process that involves the endoplasmic reticulum (ER), Golgi apparatus, and eventually the plasma membrane. Alternatively, the proteins can be secreted through secretory lysosomes, which is the path used to transport and process APP to Aβ [85]. The mechanism by which Aβ is secreted after autophagy induction is currently unknown, notwithstanding the possibility that autophagosomes may be participating in APP processing to create Aβ.

Nevertheless, in this particular situation, it has been suggested that the rise in the buildup of autophagosomes in AD has led to the expulsion of Aβ into the area outside cells, aiding the formation of Aβ [286]. Dysfunction of lysosomes within the cell can result in the buildup of Aβ inside the cell, which contributes to the series of occurrences that trigger neurodegeneration [37, 85, 287]. Enhancing the autophagic process in mouse models of AD prevented neurodegeneration and halted the deterioration in cognitive function, which was associated with an elevation in the removal of Aβ [140, 288, 289]. As an illustration, the targeted increase in the antiaging hormone Klotho in the brains of mice with the APP/PS1 genetic modification enhanced the removal of Aβ through autophagy and enhanced cognition [290]. The KL‐vS variant of the KLOTHO gene in humans, which leads to higher levels of Klotho in the bloodstream, eliminates the effects associated with the APOE ε4 gene variant. Individuals with the APOE ε4 and KL‐vS variants experience a decreased likelihood of AD.

The byproduct of APP (APP‐β‐CTF) is also associated with autophagy. AP2, an adapter protein, acts as a selective receptor for LC3 and facilitates the direct transport of APP‐β‐CTF to autophagosomes, reducing the formation of Aβ1‐42. APP‐β‐CTF elimination through AP2 binding is additionally facilitated by phosphatidylinositol binding clathrin assembly protein (PICALM), a recognized binding associate of AP2 implicated in clathrin‐mediated endocytosis [291]. AD GWAS provided convincing proof of a connection between AD and PICALM variations, indicating that PICALM plays an essential part in eliminating APP‐β‐CTF and that variants in its location are associated with a greater likelihood of AD [292, 293]. The level of PICALM is decreased in the brains of individuals with AD, and this decrease is associated with the production of Aβ and the buildup of pTau [291, 294]. PICALM has a role in controlling the creation of autophagosomes and their merging with lysosomes by influencing the activity of NSF attachment protein receptors (SNAREs), such as VAMP2 and VAMP8 [294].

The function of autophagy in maintaining Tau levels is demonstrated by the buildup of pTau and the development of neurodegeneration in mice with neuronal loss of Atg7 [103]. The relationship between the UPS and autophagy, facilitated by p62 as a mediator, has been extensively examined in Tau clearance [295]. The inhibition of p62, either alone or in conjunction with other genetic variables such as the APOE ε4 allele, led to alterations in the structure and solubility of Tau, resulting in its impaired clearance [296]. Inducing autophagy has an impact on tauopathies. Rapamycin decreased Tau phosphorylation in Tau mutant mice.

In contrast, TSC2‐KO animals, which have a genetic modification that causes constant activation of mTOR, showed increased levels of Tau and phosphorylation of Tau [104]. By utilizing mTOR antagonists with greater potency than mTOR, researchers could see a reduction in Tau phosphorylation and insoluble Tau in neural cells generated from patient iPSCs containing a Tau mutation [105]. In AD mouse models, the NDP52 identified pTau [106]. The chemical from tea extract triggered an increase in NDP52 expression, leading to the clearance of pTau in cultured neurons [107]. Dysfunctional autophagy results in the buildup of Aβ and Tau, which then hinder autophagy, commencing a detrimental loop that may ultimately end in AD.

## 7 Therapeutic Approaches Targeting Autophagy and Senescence

Although AD is widespread, treatment has not yet been found. Clinical studies investigating drugs targeting Aβ reduction have had mostly unsatisfactory outcomes. However, the US FDA recently approved the anti‐Aβ antibody aducanumab. Cellular senescence is becoming more acknowledged as a significant variable in the development of AD, which makes senotherapies a promising and appealing treatment option. Senolytic therapies, which target and eliminate SCs, have demonstrated encouraging outcomes in mouse models of neurodegeneration caused by tau and AD [207, 211, 215]. These interventions are now progressing to the clinical phase, with two scheduled clinical studies (NCT04785300i ALSENLITE and NCT04685590ii SToMP‐AD) set to evaluate the curative efficacy of the senolytic combination of D + Q in older adults with MCI. ALSENLITE is pilot research that investigates the intermittent use of dasatinib (Phase I) and quercetin (Phase II) in an open‐label manner.

On the other hand, SToMP‐AD is a Phase II experiment conducted at many sites, which is randomized, double‐masked, and placebo‐controlled. This investigation aims to assess the safety and effectiveness of the treatment in older persons with MCI or EOAD. Nevertheless, to prevent any possible adverse consequences of removing SCs, it is advisable to explore alternate senotherapeutic methods for regulating neuroinflammation [297, 298]. One such strategy might include implementing tactics that specifically target the suppression of the SASP. Furthermore, SCs exhibit diverse characteristics and constantly change, like their related secretory phenotype [299, 300, 301]. Therefore, it is crucial to clarify the specific cell types that experience senescence in AD and the chronology and characteristics of the SASP. Collaborating to create a tissue‐specific SC atlas is crucial and will be a valuable tool for evaluating the treatment possibilities of upcoming methods [302]. While several medications have demonstrated potential in mouse models, it is crucial to acknowledge that most have been unsuccessful in human experiments. This emphasizes the requirement to use models based on human cells when exploring potential ad 304–307 treatments.

The correlation between senescence and processes that induce AD is extensively demonstrated. Senescence indicators have been detected among people with neurological disorders. Furthermore, research has demonstrated that SCs that produce the cell cycle inhibiting protein p16 substantially promote age‐associated tissue degeneration and reduce the overall lifespan of healthy mice [153]. Bussian et al. established a causal relationship between the buildup of SCs and the degeneration of neurons linked with cognition. In a mouse model of NDs dependent on tau, researchers demonstrated the accumulation of p16‐positive SAs and microglial cells. After removing these cells, additional variables associated with AD showed improvement, such as gliosis, buildup of hTau, and deterioration of cortical and hippocampal neurons. Therefore, existing research indicates a connection between the cellular process and other ARDs, including AD, atherosclerosis, and osteoarthritis [215].

Increasing and ongoing SCs not only adversely impact the prognosis of NDs, but they have also been recognized as a detrimental factor in additional ARDs. They are clearing p16‐positive cells in mice, leading to a delay in the advancement of illnesses and a reduction in age‐related decline in many organs. In mice experiments, the targeted removal of specific genes and the associated elimination of SCs resulted in an extended longevity period and did not cause any noticeable adverse outcomes [153]. While it is not possible to remove specific genes in people, the use of senolytic medicines to eliminate SCs through pharmacological means shows potential as a means of therapy. Zhu et al. introduced the first senolytic medicine, which is a type of medication that explicitly triggers PCD in SCs [303]. Subsequently, other prospective individuals have been recognized. Some noteworthy instances are discussed.

Navitoclax binds to the inhibiting domains of proteins of the BCL‐2. BCL‐2 is considered a proto‐oncogene, which promotes mechanisms that prevent cell death (antiapoptotic) and inhibits the removal of SCs [304]. Navitoclax has demonstrated the ability to suppress the antiapoptotic activity of cells and facilitate the elimination of SCs, both in laboratory settings (in vitro) and in living organisms (in vivo) [305]. Bussian et al. demonstrated that giving navitoclax to mice with excess p16‐positive cells produced comparable outcomes to completely removing the related gene. The administration of the drug inhibited the expression of genes linked with senescence and reduced the phosphorylation of tau [215].

Dasatinib is a diminutive compound that is a tyrosine‐kinase antagonist in managing leukemia [306]. Quercetin is a polyphenol chemical, specifically a flavonol, commonly found in various fruits, vegetables, and plants. It is recognized for its antioxidant and tumor‐inhibitory characteristics [307]. Both drugs have been authorized by the FDA and are considered safe for human consumption. Zhu et al. employed a mechanism‐based strategy to pick the chemicals, which differs from the typical random high‐throughput screening method to develop drugs. They utilized the features of the compounds that were previously identified. D + Q has specific interactions with distinct cell types, such as mouse embryonic fibroblasts and human ECs. These two compounds had to be paired together to achieve substantial outcomes, as demonstrated in studies [303, 308, 309]. Another intriguing discovery in the investigation of D + Q is that they appear to demonstrate their senolytic capabilities even when given intermittently. Although the chemical is eliminated from the body relatively quickly, with an average half‐life of a few hours, a single dose still shows a senolytic effect for a minimum of 7 months. The regularity of delivery depends on the factors that cause cellular senescence [303, 308, 310]. In addition to the significant clinical promise of senolytic medicines, some hurdles must be overcome to fully utilize their therapeutic benefits. Given the lack of direct biomarkers for SCs, it is imperative to create accurate indicators to fully exploit the beneficial effect of senolytics.

Senolytic treatments can decelerate the advancement of AD and other aging‐related NDs [311, 312, 313, 314]. Multiple clinical trials are underway to study the safety and effectiveness of senolytic medicines, such as D + Q, for treating AD and other ARDs [312, 315].

Recent technological advancements such as single‐cell RNA sequencing (scRNA‐seq) and spatial transcriptomics have provided unprecedented resolution into cellular heterogeneity within the AD brain. These tools enable the dissection of cell‐type‐specific autophagy and senescence signatures across disease stages and brain regions [316]. For instance, scRNA‐seq has revealed microglial subpopulations with differential senescence profiles in AD [317], while spatial transcriptomics has allowed mapping of SAs in proximity to Aβ plaques and tau tangles [318]. Such technologies hold promise in uncovering early cellular dysfunctions and guiding precision‐targeted interventions against the autophagy‐senescence axis.

## 8 Conclusions

This thorough analysis highlights the crucial functions of defective autophagy and cellular senescence in advancing AD. The complex interaction between these events worsens the degeneration of neurons, leading to AD characteristics such as the formation of Aβ and NFTs made up of tau proteins that have been excessively phosphorylated. Autophagy is greatly disturbed in AD, resulting in the buildup of faulty proteins and organelles. This dysfunction is exacerbated by lysosomal deviations resulting in the buildup of Aβ and tau within cells. Cellular senescence also significantly participates in the progression of AD by producing a harmful milieu that speeds up the process of nerve cell degeneration. Potential treatments aimed at manipulating autophagy and senescence show potential for the management of AD. Pharmacological treatments, including rapamycin, metformin, and resveratrol, have been proven effective in decreasing Aβ and tau levels in preclinical animals by enhancing autophagy.

Similarly, senolytic treatments, which target the specific elimination of SCs, have been shown to decrease Aβ and tau pathology, along with neuroinflammation, in animal models. Nevertheless, the intricacy of these procedures requires a subtle strategy, as an overabundance of autophagy stimulation or extensive removal of SCs could result in harmful consequences. Subsequent investigations should prioritize the improvement of these treatments to reinstate regular autophagic activity and eradicate SCs without inducing any negative consequences. Important areas for additional research involve enhancing the ability to break down lysosomes, comprehending the molecular mechanisms that connect Aβ and tau disease to cellular senescence, and creating more accurate indicators to detect and measure SCs in living organisms. By enhancing our comprehension of the underlying processes that cause AD, we can lay the groundwork for innovative therapies that have the potential to hinder or undo the incapacitating consequences of these NDs in the aging brain.

To develop an effective treatment approach for AD, it is crucial to address the dual difficulties of defective autophagy and cellular senescence. Future studies should prioritize improving these methods and comprehending their combined impacts to facilitate the development of innovative therapies that can stop or reverse the advancement of these incapacitating NDs. An effective approach is the exploration of combinatory medicines that can increase autophagy and destroy SCs concurrently. Future therapeutic tactics are expected to rely heavily on precision medicine approaches, which customize medicines based on individual patients' genetic and molecular profiles. In addition, investigating the possibility of lifestyle interventions, such as changes in food and physical activity, which have been proven to affect autophagy and cellular senescence, may provide other techniques to supplement pharmacological treatments. By incorporating these diverse methods, we can greatly enhance our capacity to fight against AD and enhance the well‐being of those affected.

## Author Contributions

**Md Sadique Hussain, Neetu Agrawal, and Baby Ilma:** conceptualization, data curation, methodology, visualization, writing – original draft. **Rekha M M, Priya Priyadarshini Nayak:** methodology, visualization, writing – original draft. **Mandeep Kaur, Anil Khachi:** data curation, methodology, writing – original – draft. **Kavita Goyal, Arcot Rekha:** methodology, writing – original draft. **Saurabh Gupta:** writing – review and editing. **Gaurav Gupta, Kamal Dua:** conceptualization, data curation, methodology, writing – review and editing, funding acquisition.

## Ethics Statement

The authors have nothing to report.

## Consent

Consent to publish has been obtained from all authors.

## Conflicts of Interest

The authors declare no conflicts of interest.

## Data Availability

Data sharing is not applicable to this article as no new data were created or analyzed in this study.
